# A practical method for predicting frequent use of emergency department care using routinely available electronic registration data

**DOI:** 10.1186/s12873-016-0076-3

**Published:** 2016-02-09

**Authors:** Jianmin Wu, Shaun J. Grannis, Huiping Xu, John T. Finnell

**Affiliations:** Regenstrief Institute, Inc., 1101 West 10th Street, Indianapolis, IN 46202 USA; Department of Biostatistics, Indiana University School of Public Health and School of Medicine, Indianapolis, IN 46202 USA

**Keywords:** Emergency department, Registration data, Prediction, Frequent use

## Abstract

**Background:**

Accurately predicting future frequent emergency department (ED) utilization can support a case management approach and ultimately reduce health care costs. This study assesses the feasibility of using routinely collected registration data to predict future frequent ED visits.

**Method:**

Using routinely collected registration data in the state of Indiana, U.S.A., from 2008, we developed multivariable logistic regression models to predict frequent ED visits in the subsequent two years. We assessed the model’s accuracy using Receiver Operating Characteristic (ROC) curves, sensitivity, and positive predictive value (PPV).

**Results:**

Strong predictors of frequent ED visits included age between 25 and 44 years, female gender, close proximity to the ED (less than 5 miles traveling distance), total visits in the baseline year, and respiratory and dental chief complaint syndromes. The area under ROC curve (AUC) ranged from 0.83 to 0.92 for models predicting patients with 8 or more visits to 16 or more visits in the subsequent two years, suggesting acceptable discrimination. With 25 % sensitivity, the model predicting frequent ED use as defined as 16 or more visits in 2009 and 2010 had a PPV of 59.5 % and specificity of 99.9 %. The “adjusted” PPV of this model, which includes patients having 8 or more visits, is 81.9 %.

**Conclusion:**

We demonstrate a strong association between predictor variables present in registration data and frequent ED use. The algorithm’s performance characteristics suggest that it is technically feasible to use routinely collected registration data to predict future frequent ED use.

## Background

With increasing medical costs, health care reformers and policy makers have turned to emergency department (ED) utilization as a potential source for cost savings. A relatively small number of patients, often called “frequent” or “high ED users”, have been an increasing focus because of their disproportionate share of ED visits and cost. When defined as 4 or more ED visits per year, frequent users accounted for 4.5 to 8 % of all ED patients and contributed 21 to 28 % of all ED visits [1]. Prior interventions targeting frequent users did not achieve universally positive outcomes although some studies demonstrate reduced ED use [2–6]. A clear framework including a consensus-based definition of frequent users and methods to accurately and consistently identify this population may improve the effectiveness of care management interventions.

Currently, however, a standardized definition for frequent ED users remains elusive. A single visit threshold has been used to differentiate frequent users from low ED users, and the visit threshold varies from as few as 3 to 12 or more annual visits, often without a clear rationale for the visit cut-point [7–13]. Further, the majority of prior studies on frequent ED users focused on identifying existing frequent ED users [7, 9, 11, 14], which is problematic as most frequent users in a given year will not remain frequent users in the next year. It has been shown that an individual who has 4 or more visits in a given year was only 28 to 38 % likely to be a frequent user the next year [1]. Fertel et al. also showed that highly frequent use occurs for only a minority of ED patients, and then only for a discrete period [15]. Roland et al. pointed out that the “regression to mean” phenomenon should not be ignored when evaluating interventions for frequent ED users [16]. Therefore, blindly targeting most current frequent ED users for future interventions is inefficient because their heavy use of ED services may decrease without intervention. Since health care resources are limited, it is essential that interventions target patients whose heavy ED use will likely persist. Thus, the capacity to predict patients who are likely to sustain frequent future ED utilization can help address this problem by identifying patients who are most likely to generate future heavy ED use and costs.

Previously we reported that 2.8 million patients from 96 EDs in the state of Indiana within United States generated 7.4 million ED visits from 2008 to 2010, and the average number of visits was 2.6 visits per patient [17]. We found that patients cross over to other ED institutions with great frequency, and about 3.3 % of the patients made more than 10 visits to Indiana EDs from 2008 to 2010 [17]. In this study, we explore whether specific features contained within routinely gathered registration data could meaningfully predict a patient’s future ED utilization. If these features accurately predict future frequent ED users, then we can more effectively, target limited health care resources on this group, maximizing the benefit of the intervention. The purpose of this study is to assess the feasibility of using routinely gathered registration data to predict patients who will visit ED’s with high frequency.

## Methods

This study was approved by Indiana State Department of Health (ISDH) data release committee and the Indiana University Institutional review board (USA).

### Datasets

Data collected for this study were derived from the original Health Level-7 (HL7) version 2 registration transactions for ED encounters from 96 institutions participating in the Indiana Public Health Emergency Surveillance System (PHESS) between January 1, 2008 and December 31, 2010. The data is not publically available but can be accessed through the Regenstrief Institute Data Core (https://www.regenstrief.org/hsr/research-programs/rcher/data-core/).

The processes for preparing ED encounter data as well as the details for each step were presented in our previous paper [17]. Briefly, registration transactions were processed to ensure each transaction was unique and contained valid ED encounter data according to PHESS requirements and a set of heuristics drawn from Regenstrief’s long-term real-world experience operating a health information exchange. Unique ED encounters were established using data elements including person, place and time. The specific fields included [1] healthcare institution (HL7 MSH-4), [2] ED encounter date (HL7 PV1–44), and [3] medical record number (HL7 PID-3). Transactions missing any of these fields could not be definitively and uniquely identified as an encounter and were excluded from the analysis.

Unique patients were identified using various combinations of patient demographics, including social security number, last and first name, gender, date of birth, telephone number, and zip code as determined by an open-source probabilistic record linkage software package [18]. In this manner all ED encounters belonging to the same patient were linked, forming a “patient group.” A unique global patient identifier was assigned to each patient group. In total, we identified 7,447,521 unique ED encounters. Data available for analysis includes: age, sex, chief complaints, ZIP codes for patients’ address, and hospital ZIP codes. Patients’ global identifier was used to link visits across different hospital databases, including all ED visits regardless of disposition.

### Predictive model

We developed multivariable logistic regression models. Patients with at least one ED visit in 2008 were used to predict ED visits in the years of 2009 and 2010. Patients who died before January 1, 2009 or had missing values in one or more covariates were excluded (<4.30 %). The final sample size was 1,272,367 patients. All variables were summarized at the patient level for model development.

### Covariates

All covariates were determined based on the ED utilization data in 2008.

*Age:* age was determined at the time of the first ED visit, and divided into six subgroups: <5, 5–14, 15–24, 25–44, 45–64 and > =65 years.

*Sex:* male and female;

*Visits in 2008*: the total number of ED visits made in 2008 for each patient;

*Chief complaints*: the chief complaint syndromes were grouped into 11 categories: respiratory, gastrointestinal (GI), undifferentiated infection (UDI), influenza-like illness (ILI), lymphatic, skin, neurological, pain, dental, alcohol and musculoskeletal syndromes. These categories were used by other surveillance programs with slight modification [19–21]. Chief complaints that could not be grouped into the above 11 syndromes were assigned to “unclassified”. The categories were then reviewed by two physicians (Grannis S, Finnel JT) and an epidemiologist. For each patient, the proportion of each chief complaint syndrome is determined through dividing the number of ED visits with a specific syndrome by the total number of ED visits that the patient had in 2008. Since one ED visit may have more than one syndrome, these percentages do not add up to 100 %.

*Zip code centroid straight-line distances*: The Perl library Geo::Distance was used to calculate the straight-line distances between geographic points from patients’ home to hospital based on zip code centroids of patient’s home address and hospital address. Distance was then grouped into 3 categories: <=5 miles, 5–20 miles and >20 miles. Since one patient may have multiple ED visits with different distance, we determined the proportion of ED visits falling into each of the three categories by dividing the number of ED visits with a specific distance category by the total number of ED visits that a patient made in 2008. Because the proportions for each of these three distance categories add up to 100 %, only two categories (<5 miles and >20 miles) were included in the analytic model.

### Study outcome

The outcome was measured as dichotomized variable (frequent versus low ED user). Frequent ED users were investigated by using visit cut-points ranging from 8 to 16 visits over a two-year period (between 2009 and 2010). One model was fit for each cut point. Patients were defined as frequent ED users if their ED visits were equal to or higher than the visit cut-point, and were otherwise defined as low ED users.

### Model performance evaluation

The model’s performance was assessed for discrimination using the Receiver Operating Characteristic (ROC) curves. We balanced the goal of identifying all frequent ED utilizers with the intervention cost of incorrectly identifying frequent ED users by selecting a fixed sensitivity of 25 % to minimize the false positive rate. We then evaluated the specificity and positive predictive value (PPV) for each model at fixed sensitivity of 25 %. We also combined the false positive (FP) patients who had 8 or more visits with the true positive (TP) patients to obtain the “adjusted” positive cohort. The “adjusted” PPV was determined by dividing the “adjusted” positive group by the sum of TP and FP. Statistical analyses were conducted using SAS version 9.3 (SAS Corporation; Cary, North Carolina).

## Results

### ED utilization and patients’ distribution by year

**Fig. 1:**
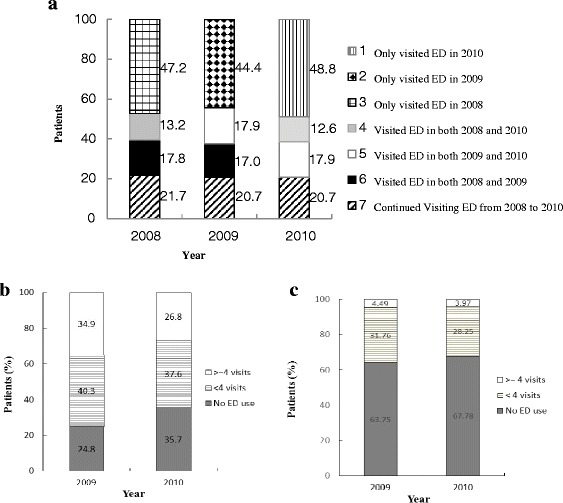
**a**. Distribution of patients stratified by year, 2008 to 2010; **b**. Emergency department utilization in 2009 and 2010 for patients with 4 or more visits in 2008; **c**. Emergency department utilization in 2009 and 2010 for patients with fewer visits less than 4 in 2008

During the 3-year study period, 2.8 million patients generated 7.4 million ED visits. Of patients with ED visits in 2008, about one-fifth came back to the ED in both 2009 and 2010 and one-third of them returned either in 2009 or in 2010. Almost half did not present to ED in the following two years (Fig. 1a). Interestingly, patients who made 4 or more visits in 2008 showed a similar pattern. 8.5 % of all 2008 patients had 4 or more visits in 2008. But only 35 % and 27 % of them continued to have 4 or more visits in 2009 and 2010 respectively. The majority returned to low or no ED use in the subsequent two years (Fig. 1b). Interestingly, about 4 % of patients with less than 4 visits in 2008 increased their ED utilization to 4 or more visits either in 2009 or 2010 (Fig. 1c).

### Characteristics of ED patients

Patients aged 25 to 44 years were the largest group and accounted for ~29 % of the total visits and ~26 % of total patients. The second largest age group were 44 to 65 years and contributed ~20 % of the total visits. Patients aged 5 to 14 years had the least number of visits and accounted for 8 to 9 % of total visits in each year, respectively. More than 53 % of patients were female and they accounted for 55 % of visits.

The distribution of patients with ED visits equal to or greater than 4, 8, and 16 annually is presented in Table 1. Patients with 4 or more visits per year accounted for 30 to 45 % of the total visits each year. Although the patients who had 16 visits annually only accounted for 0.3 % of the total patients, they contributed almost 4 % of the total visits.

**Table 1:** Distribution of patients and ED visits by visit cut-points in each year

	Year (Pat. No.)	Visits cut-point		
		> = 4	> = 8	> = 16
Patients (%)	2008 (n = 1329645)	8.5	1.6	0.3
	2009 (n = 1396313)	9.2	1.7	0.3
	2010 (n = 1397338)	8.9	1.6	0.3
Visits (%)	2008 (n = 2367399)	29.8	11.0	3.7
	2009 (n = 2551881)	31.3	11.6	3.9
	2010 (n = 2528241)	45.1	21.0	3.9

The profile of ED visits by the zip code centroid straight-line distance between home and hospital in each year is very similar. More than 60 % of ED visits were associated with a distance of 5 miles or less. About 30 % of visits had a distance between 5 and 20 miles, and 8 % of visits had distance of more than 20 miles (Table 2).

**Table 2:** Distribution of ED visits by travel distance and chief complaints in each year, 2008 to 2010

Characteristics	2008 (%)	2009 (%)	2010 (%)
Travel distance (miles)			
<=5	60.1	60	60.1
5–20	30.2	30.7	30.3
>20	8.4	8.1	8.3
Missing	1.4	1.2	1.3
Chief complaint^a^			
Respiratory	23.1	24.6	22.0
Gastrointestinal	17.1	17.6	17.7
Neurologic	11.3	11.2	11.2
Skin	4.6	4.4	4.7
UDI	9	10.5	8.8
Lymphatic	3	3.0	3.0
ILI	18.8	20.7	20.9
Dental	1.8	1.8	1.8
Pain	41.5	40.3	40.8
Musculoskeletal	28.7	27.5	27.7
Alcohol	0.5	0.5	0.6
Unclassified	15.7	14.8	15.4
Missing	1.34	1.47	3.41

^a^Because one visit may have multiple syndromes, the total percentage of chief complaints is more than 100 %

Table 2 captures chief complaint distributions. “Pain” was the most common chief complaint category, accounting for more than 40 % of total visits, while the “musculoskeletal” category contributed ~28 % of total ED visits. “Respiratory” and “gastrointestinal” syndrome categories accounted for more than 23 % and 17 % of total visits, respectively. The “alcohol” accounted for 0.5 % of total visits each year. Nearly 15 % of visits were grouped into the “unclassified” category.

### Multivariable logistic regression model predicting frequent use of ED care

**Fig. 2:**
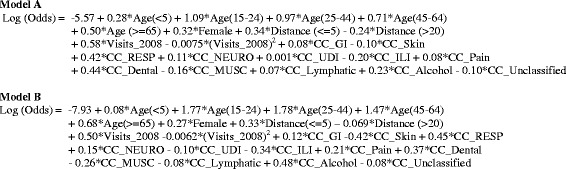
Equations for model predicting frequent emergency department (ED) use as defined as 8 or more visits (**a**) and model predicting frequent ED use as defined as 16 or more visits in the subsequent two years (**b**). Distance (<=5): the straight-line distances between geographic points from patients’ home to hospital less than 5 miles; Distance (>20): the straight-line distances between geographic points from patients’ home to hospital greater than 20 miles; CC: chief complaints; GI: gastrointestinal; RESP: respiratory; NEURO: neurological; UDI: undifferentiated infection; ILI: influenza-like illness and MUSC: musculoskeletal

We developed logistic regression models to predict the likelihood for a patient to be a frequent ED user in years 2009 and 2010 based on the 2008 registration data. Table 3 presents the patients’ characteristics used in the models stratified by number of visits in 2009 and 2010. Overall, common characteristics of frequent ED users include age between 15 and 44 years, female gender, and utilizing ED care frequently in the baseline year. They commonly report “pain”, “musculoskeletal”, “respiratory”, and/or “gastrointestinal” syndromes. More than 77 % of frequent ED users had one or more visits with a travel distance equal to or less than 5 miles. Table 4 shows the results from two logistic regression models predicting frequent use of ED with 8 or more visits, and frequent use of ED with 16 or more visits, respectively. Age, sex, zip code centroid straight-line distance between home and hospital, and specific chief complaints, including respiratory, dental, and alcohol syndromes were predictive for frequent use of the emergency room in 2009 and 2010. The variable representing number of visits in 2008 plus a quadratic term to account for some non-linearity in age associations led to a better fit of the model, and was highly predictive for frequent use of ED the following two years. The significance of the predictors is consistent across these models, and the coefficients for most of the predictors revealed a similar profile. The equations for these two logistic regression models are summarized in Fig. 2.

**Table 3:** Distribution of predictors in 2008 stratified by number of visits in 2009 and 2010

	Visits in 2009 & 2010				Total (Patients No.)
	0	1–7	8–15	> = 16	
Year 2008 patients (No.)	587523	623355	47782	13707	1272367
Age (Years) (Patients, %)					
15–24	44.1	49.2	5.2	1.5	206230
25–44	45.9	47.4	4.8	1.9	346349
45–64	50.5	44.9	3.5	1.1	262691
5–14	51.6	46.8	1.4	0.1	137222
<5	43.7	53.6	2.5	0.2	131743
> = 65	40.6	55.7	3.3	0.4	188132
Sex (Patients, %)					
Male	49.6	46.6	2.9	0.8	590065
Female	43.2	51.1	4.5	1.3	682302
Visits in 2008 (Patients, %)					
<4	49.2	48.2	2.3	0.3	1167719
4–7	14.6	62.8	17.8	4.8	85359
8–15	6.2	37.9	31.2	24.7	15964
> = 16	2.7	13.1	19.9	64.3	3325
Chief complaints in 2008 (Visits, %)					
RESP	62.9	28.7	5.1	3.3	976602
GI	32.2	50.6	9.6	7.6	390479
NEURO	34.4	51.6	8.4	5.6	248617
SKIN	35.8	54.0	7.2	3.1	105329
LYMP	33.3	52.8	8.9	5.1	65627
UDI	33.5	56.3	7.0	3.2	207325
PAIN	34.7	49.4	9.1	6.9	953772
ILI	33.0	54.3	8.1	4.6	429244
DENTAL	24.7	51.5	13.8	10.0	41990
MUSC	37.8	49.2	7.9	5.1	654713
ETOH	33.7	43.8	11.1	11.4	12286
OTHER	37.3	50.0	7.8	4.8	353451
Distance (Miles) (Visits, %)					
<=5	34.8	63.1	1.6	0.4	952546
5–20	42.7	55.9	0.8	0.6	618869
>20	56.5	41.9	0.4	1.2	173725

**Table 4:** Multivariable logistic regression models predicting frequent ED users having > = 8 visits and frequent ED users having > =16 visits in 2009 and 2010

Predicting variable	> = 8 visits			> = 16 visits		
	Coefficient	Standard error	*p* value	Coefficient	Standard error	*p* value
Age (year)						
5–14	Ref					
<5	0.2823	0.0292	<.0001	0.0763	0.1024	0.4562
15–24	1.0897	0.0248	<.0001	1.7732	0.0801	<.0001
25–44	0.9673	0.0241	<.0001	1.7804	0.079	<.0001
45–64	0.7146	0.0252	<.0001	1.4661	0.0805	<.0001
> = 65	0.5033	0.0266	<.0001	0.6751	0.0864	<.0001
Visits in 2008	0.5829	0.00243	<.0001	0.5048	0.00319	<.0001
(Visits in 2008)^2^	−0.0075	0.00011	<.0001	−0.0062	0.00011	<.0001
Sex (ref. = “Male”)						
Male	Ref.					
Female	0.3173	0.00978	<.0001	0.2663	0.0212	<.0001
Travel Distance (miles)						
5–20	Ref.					
<=5	0.344	0.0115	<.0001	0.3263	0.0259	<.0001
>20	−0.2396	0.023	<.0001	−0.0685	0.0501	0.171
Chief Complaints						
Respiratory	0.4202	0.0203	<.0001	0.4527	0.0487	<.0001
Gastrointestinal	0.0783	0.019	<.0001	0.1195	0.0437	0.0062
Neurologic	0.1084	0.021	<.0001	0.1487	0.049	0.0024
Skin	−0.0983	0.0317	0.002	−0.4246	0.0858	<.0001
UDI	0.00071	0.0254	0.9777	−0.0999	0.0648	0.123
Lymphatic	0.066	0.0361	0.0679	−0.0848	0.0896	0.344
ILI	−0.2013	0.0201	<.0001	−0.3398	0.0489	<.0001
Dental	0.4419	0.0393	<.0001	0.372	0.0843	<.0001
Pain	0.081	0.0153	<.0001	0.2139	0.0359	<.0001
Musculoskeletal	−0.1625	0.0186	<.0001	−0.2645	0.0434	<.0001
Alcohol	0.2296	0.0753	0.0023	0.4842	0.1447	0.0008
Unclassified	−0.1018	0.0241	<.0001	−0.0771	0.0574	0.1789

We next evaluated the model’s performance using ROC curves, specificity, and PPV (Table 5). The model predicting frequent ED use with 8 or more visits in the subsequent two years showed satisfactory discriminating power with an AUC of 0.84 (Model No.1). The model predicting frequent ED use as defined as 16 or more visits in the subsequent two years showed better discrimination, with AUC of 0.92 (Model No. 9). We also explored patient characteristics among the false positive (FP) and false negative (FN) groups. Interestingly, patients in the FP group had an equal number of visits in the 2008 baseline year when compared to the true positive (TP) patients (Mean visits of FP vs TP: 11.0 ± 4.3 vs 14.1 ± 7.9 for Model No. 1; 18.9 ± 6.8 vs 22.8 ± 9.8 for Model No. 9). However, when compared with the TP cohort, the FP patients yielded a lower proportion aged 15–24 year, and a higher proportion > =65 years, fewer were female, and there was a lower proportion of “respiratory” chief complains. Regarding the FN group, their total number of visit in the baseline year was comparable to the true negative (TN) group (Mean visits of FN vs TN: 3.3 ± 3.2 vs 1.5 ± 1.0 for Model No. 1; 6.08 ± 6.3 vs 1.7 ± 1.3 for Model No. 9). The FN group also exhibited a larger proportion of “respiratory” and “alcohol” complaints; more were aged 25–44 years, and more were associated with a travel distance within 5 miles when compared to the TN group. Finally, we evaluated the specificity and PPV of the models at 25 % sensitivity. Model No. 9 which predicts frequent ED use as defined as 16 or more visits in 2009 and 2010, revealed a PPV of 59.5 % and specificity of 99.9 %. A significant proportion of FP patients visited the ED repeatedly although they did not meet the specific threshold of frequent ED use. For example, 55.3 % of FP patients had 8 or more visits in Model No. 9, and the “adjusted” PPV, which included patients having 8 or more visits in the subsequent two years as positives targets, was 81.9 %.

**Table 5:** Model evaluation

		Multivariable Logistic Regression Models								
Model No.		1	2	3	4	5	6	7	8	9
No. of visits constituting ‘frequent use’		> = 8	> = 9	> = 10	> = 11	> = 12	> = 13	> = 14	> = 15	> = 16
Area under ROC curve (AUC)		0.84	0.85	0.87	0.88	0.89	0.89	0.90	0.91	0.92
With sensitivity < =25 %, probability > 0.5										
PPV (%)		64.5	63.9	63.4	62.9	62.4	61.3	60.8	60.6	59.5
Specificity (%)		99.5	99.6	99.7	99.7	99.8	99.8	99.8	99.8	99.9
False positive patients	Total No.	5883	4923	4125	3447	2974	2610	2273	2007	1805
	> = 8 visits (No.)	0	565	843	1021	1071	1103	1077	1038	998
Adjusted PPV for patients with > =8 visits in subsequent two years (%)		64.5	68	70.9	73.9	75.9	77.7	79.4	81	81.9

## Discussion

The primary goals of this study were to evaluate the feasibility of using routinely available registration data to predict patients likely to use ED services frequently in the future and to develop strategies for improving the accuracy and efficiency of detecting frequent ED users. We demonstrate a strong association between predictor variables present in routine registration data and frequent ED use. The algorithm’s performance characteristics suggest that it is technically feasible to use routinely collected registration data to predict such use, and the model’s observed prediction accuracy may support identifying and intervening upon frequent ED users. Thus, such models may support more effective targeting of limited health care resources to patients who may maximally benefit from intervention.

Much of the literature studying frequent ED utilization has substantial limitations, which our study sought to address. First, some published studies used data from a limited number of ED’s and thus their broad generalizability is unclear [7–12, 22, 23]. Although several statewide studies in United States explored ED visits across age, gender, health insurance groups and clinical characteristics between frequent and in-frequent ED users, most were descriptive in nature and few applied prediction models to identify frequent ED users [24–27]. Second, some studies used survey or interview data and the quality and reliability of such data can be affected by survey response rates [8–10]. Further, the cost, time and other resources involved in the interview may be prohibitive. Third, some studies focused on specific cohorts such as asthmatics or the elderly, and this limits the ability of policy makers and providers to determine whether unifying factors that could be targeted for intervention exist amongst a more general population of patients with frequent ED utilization. Lastly and most importantly, in many cases researchers focused on identifying existing frequent ED users instead of predicting future frequent ED utilization [7, 9, 11, 14]. As shown in ours and others studies [1, 15, 16], most patients do not remain frequent ED users over time and many naturally reduce their ED use without intervention (regression toward mean). Thus, predicting patients who are likely to sustain future frequent ED utilization will be necessary for improving the health of this vulnerable patient group.

Developing algorithms that accurately identify patients who are likely to frequently visit ED’s in subsequent years is a first step toward developing potential interventions to mitigate overuse. However, few studies have leveraged any approach or method to identify future frequent ED users [4,28–31]. In those studies, frequent ED users were defined with a threshold number of ED visits, e.g. 3 to 10 ED visits within the 12 months prior to the study period. In addition, the majority of the comparative cohort studies used a pre-and post-intervention design, where the population exposed to the intervention served as their own historical control groups, without recognizing the regression toward mean phenomenon, which might incorrectly inflate the effectiveness of interventions.

In our study, we developed a practical approach to predict future frequent ED users. The model predicting patients with 8 or more visits in the subsequent two years demonstrated reasonable discriminative power with an AUC of 0.84. As the threshold defining ‘frequent use’ increases, the corresponding AUC also increased. The model predicting frequent ED use of 16 or more visits in the subsequent two years showed good discrimination, with an AUC of 0.92. Strong predictor variables included visits in the baseline year, age, sex, zipcode centroid straight-line distance between home and hospital, and specific chief complaints, including respiratory, dental and alcohol syndromes. When comparing false positives to true positives and false negatives to true negatives, respectively, we noted that the variable “Number of visits in the baseline year” were very close, indicating that patients’ other features contained within routinely gathered registration data contributed additional discriminating power.

If the algorithm incorrectly flags patients as frequent utilizers, the resulting inefficiencies may offset potential savings from subsequent reduced ED utilization. Considering the trade-offs between (a) identifying the maximal number of subjects who are truly frequent ED use patients and (b) minimizing subjects incorrectly flagged as frequent ED use patients, we aimed to balance the cost of incorrectly identifying frequent ED patients by setting the prediction model’s sensitivity at 25 %. Although the models had PPVs around 60 %, a significant proportion of false positive patients actually had more than 8 ED visits in two years. The adjusted PPV for patients having 8 or more visits in the model that predicting frequent ED users as 16 or more visits is 81.9 %. To our knowledge, this is the first study to employ routine registration data to develop predictive algorithm to predict future frequent ED use. The prediction accuracy strongly suggests that it is feasible to apply routinely collected registration data for future frequent ED utilization prediction.

Limitations of our study include the following: First, we lacked comprehensive population level data for persons who did not use the emergency department. Therefore our analysis is limited to characterizing those individuals who present to emergency departments. Second, we did not include data such as patients’ socioeconomic status, since that data is not routinely captured in ED registration data. Third, the applicability of our model to ED registration data from other sites is not assessed, and the predictive performance of the models might be overrated. In the future, we seek to validate this approach against other datasets in a geographically distinct region. Finally, we only evaluated models with 25 % sensitivity as we aimed to balance the cost of ED utilization and the intervention support cost from incorrectly identified frequent ED users.

## Conclusions

We demonstrate a strong association between predictor variables present in routine registration data and frequent ED use. This analysis suggests that it is technically feasible to use routinely collected registration data to identify such use, and the model’s observed prediction accuracy may support identifying and intervening to ensure health care resources will be delivered to ensure this group will maximally benefit from intervention. Future work will include validating our algorithm using data sets from other state or organizations within United States.
